# Pure Food Bill Wanted

**Published:** 1906-09

**Authors:** 


PURE FOOD BILL WANTED. — Now that Congress has at last passed a pure food law, it is in order and necessary, to make it effective, that every State should enact a similar law. We have on our statute books an excellent law, passed in 1885, but as no appropriation was ever made to carry it into effect, it has ever been, and is now, a dead letter. Now that our border and coast quarantine will soon be turned over to the United States government and all border and Gulf inspectors by the State will be discontinued, not less than half of the yearly appropriation for the Quarantine Department will be saved, and the State can well afford and may be induced to expend the necessary amount to vivify this bill, as well as to make operative the vital statistics law.

---

DR. CHAS. L. MCMANUS, of Brownsville, Texas, died in Matamoras, Mexico, August 17, ult., aged 85. He was a surgeon in the Federal army in 1846, a veteran of the Mexican war,—had been "decorated by Maximillian for meritorious services," so the dispatches say, and had been a staunch supporter of the "Red Back" twenty-one years. Dr. McManus was born in Ireland and came to America sixty years ago, settling in Brownsville (Matamoras).

